# Synthesis of a Novel Sepiolite–Ag–Propolis Nanocomposite and Its Effect on the Growth of *Aspergillus flavus*

**DOI:** 10.1155/ijm/7371265

**Published:** 2025-03-24

**Authors:** Elham Rezvannejad, Maryam Fayazi, Batool Sadeghi, Azadeh Boustan, Safa Lotfi

**Affiliations:** ^1^Department of Biotechnology, Institute of Science and High Technology and Environmental Sciences, Graduate University of Advanced Technology, Kerman, Iran; ^2^Department of Environment, Institute of Science and High Technology and Environmental Sciences, Graduate University of Advanced Technology, Kerman, Iran; ^3^Department of Medical Plant, University of Zabol, Zabol, Iran; ^4^Department of Animal Science, University of Mohaghegh Ardabili, Ardabil, Iran

**Keywords:** antifungal activity, *Aspergillus flavus*, nanocomposite, propolis, sepiolite

## Abstract

Since aflatoxin produced by *Aspergillus flavus* carries significant impacts on the livestock and poultry industries in terms of animal health and food safety. It is very important to find nonchemical antifungal agents. For this purpose, in this study, bee propolis and its nanocomposites with sepiolite and Ag nanoparticles were investigated for antifungal activity with respect to their use as safer alternatives for conventional antifungal treatments. In the present study, two newly synthesized sepiolite–propolis and sepiolite–Ag–propolis nanocomposite formulations were characterized with different analytical techniques such as XRD, TEM, FTIR, and EDAX. The antifungal potential was determined against *A. flavus* by the disc diffusion method, and MIC-MFC values were determined. The pure propolis extract had only limited antifungal activity at concentrations up to 400 mg/mL. However, prominent antifungal activities were observed for nanocomposites with propolis, sepiolite, and Ag nanoparticles, as inhibition was observed even at a low concentration of 200 mg/mL. The sepiolite–Ag–propolis nanocomposite outperformed others by increasing the diameter of inhibition zones proportionally with the increase in concentration. The finding results indicate that propolis-based nanocomposites, especially when combined with Ag nanoparticles, hold a promise for antifungal action against *A. flavus*. Further work is necessary to test their practical value in agricultural and food safety contexts.

## 1. Introduction

The natural gummy substance collected by bees from parts of plants mixed with beeswax and saliva enzymes is called propolis. Propolis mainly consists of resin (50%), pollen (5%), essential oil (10%), wax (30%), and other substances (5%) such as minerals, organic compounds, and waste. The difference between the compositions of propolis is due to the different plants and the different seasons in which the bees can collect the material. The organic compounds found in propolis are such as fatty acids, terpenoids including steroids, naphthalene, stilbene derivatives, and polyphenols [1, 2]. Various biological properties have been identified in propolis, such as antioxidant, antimicrobial, anti-inflammatory, spasmolytic, anesthetic, antidiabetic, immune-modulating, and anticancer effects [2, 3].

*Aspergillus* is a genus containing several hundred species of molds found in diverse climates around the world [4]. *Aspergillus* reproduces asexually as it does so by making spores, and this feature is common among all members of this species. Some of its species are pathogenic, and others have industrial and food applications. *Aspergillus flavus*, *Aspergillus parasiticus*, and *Aspergillus fumigatus* are pathogenic species, and *Aspergillus niger* and *Aspergillus oryzae* are beneficial species [4]. The most common species of fungus associated with aflatoxin biosynthesis is *Aspergillus flavus*, the exposure of which can cause severe damage to human and animal health. Today, one of the critical food safety issues is the contamination of food by aflatoxins, because aflatoxins have been identified in freshly harvested and processed agricultural products [5]. In the report of Schmale and Munkvold [6], the economic influence of mycotoxins included reduced crop value, yield loss, reduced animal efficiency, and additional costs because of health-related troubles in humans.

Since aflatoxins and chemicals often have adverse effects on human immunity, it is necessary to develop ways to reduce their pollution with other nonchemical alternatives. Therefore, to deliver agricultural goods to consumers in secure conditions, active formulations of natural compounds have become very important. There are several animal and plant biodegradable formatives whose antimicrobial activity has been widely reported [7].

Owing to their excellent thermal stability, high intrinsic capacity, and low cost, natural clay minerals, such as montmorillonite [8, 9], palygorskite [10], sepiolite [11–13], and kaolin [14, 15], are recognized as optimal mineral carriers in antibacterial agents. Among clay minerals, sepiolite is widely used in various fields because of its low cost, rich resources, and abundant absorption sites. In addition, sepiolite is not a health risk for people [16]. Therefore, the application of sepiolite in the antibacterial field has attracted wide attention [17–19]; however, the antimicrobial properties and mechanism of sepiolite-based antimicrobials are still being studied and discussed.

Sometimes, the incorporation of two antimicrobial materials into a formulated compound results in a greater reduction in the number of microorganisms through synergistic effects. For example, the antimicrobial agents in formulations disrupt microbial cell walls, inhibit enzyme activity, or interfere with microbial DNA replication. In addition to being applied directly on food with active antimicrobial component incorporation, these compounds can also be incorporated into a coating that allows controlled gradual release of active agents onto the food surface, maintaining antimicrobial activity over a long period [20]. This method has special value in food preservation, assisting in the control of microorganisms responsible for both spoilage and foodborne diseases. The development of nanostructured formulations has been especially promising in this regard, as their increased surface area and enhanced reactivity improve antimicrobial efficiency, providing more effective food protection [21, 22].

Hence, the objectives of this study include the morphological and physical characterization of nanostructured sepiolite formulations with propolis and Ag nanoparticles (NPs). Additionally, this study is aimed at evaluating the antifungal activity of propolis and synthesized nanocomposites against *Aspergillus flavus* under laboratory conditions.

## 2. Materials and Methods

The fungal strain *Aspergillus flavus* ATCC 5004 was obtained from the Institute of Razi Karaj. The fungal strain was placed in potato dextrose broth (PDB) culture medium for 48 h at a temperature of 37°C in a shaker incubator. After growth, the fungus was cultured in a potato dextrose agar (PDA) culture medium.

### 2.1. Preparation of Antifungal Agents

#### 2.1.1. Sepiolite Preparation

To prepare sepiolite (Iranian sepiolite sample with a particle size of ≤ 0.075 mm (200-mesh) from the Yazd region in central Iran), a suspension containing 10 g/L of sepiolite was first prepared and then stirred for 24 h. After 2 min, when the solution remained stationary, the upper solution of the suspension was separated with filter paper. To dry, the purified sepiolite sample was incubated at 105°C for 24 h [23].

Then, 10 g of sepiolite from the previous step was placed in 100 mL of 1 M nitric acid at a temperature between 70°C and 90°C for 1 h on a magnetic stirrer. To reach a neutral pH, washing with water, separation by centrifugation, and ultrasonic dispersion were performed alternately. Finally, the sediment was dried at 110°C for 24 h.

#### 2.1.2. Propolis Preparation

Freshly prepared beehive propolis was crushed and extracted twice with 70% ethanol (1:10, w:v) at room temperature for 24 h. The solution composition was dried and used in this research.

#### 2.1.3. Nanoformulation Preparation

To prepare the propolis–sepiolite nanocomposite, 0.5 g of sepiolite was added to a 70% ethanol solution containing 0.5 grams of propolis, and the mixture was stirred for 1 h at 37°C. After incubating the mixture for 24 h, it was centrifuged at 15,000 rpm, washed with distilled water, and centrifuged again to separate the components. The resulting material was then dried overnight at room temperature. Finally, the dried light brown precursor was dispersed in water to obtain the final nanocomposite suspension.

To prepare the sepiolite–Ag–propolis nanocomposite, after synthesizing the propolis-sepiolite compound as described in the previous step, 10 mL of silver nitrate solution (50 mg/L) was added dropwise to the reaction mixture under vigorous stirring. The mixture was then stirred magnetically at room temperature for 4 h to reduce the silver ions on propolis–sepiolite substrate. Thereafter, the final product was centrifuged, washed four times with ultrapure water to remove any unreacted materials, and dried overnight.

#### 2.1.4. Disk Diffusion

Before determining the MIC and MBC, the antifungal activity of the preparations was first assessed using the agar well diffusion method to screen for potential activity against *A. flavus*.

A suspension of the fungal spores was prepared by adjusting the turbidity to 0.5 McFarland standard (~1 × 10^6^ CFU/mL). One hundred microliters containing pure propolis extract was added to a tube, and then half of the solution was removed from it, added to the next tube, and continued in the same way until tube number 10. The volume of each tube was increased to 100 *μ*L with the pure culture medium. After preparing various dilutions of the extracts, molten PDA medium at 60°C was poured into plates and allowed to solidify. Wells were then created in the solidified medium, and the prepared extracts were added to these wells. A fungal inoculum of approximately 0.2 mm in size was placed at the center of each plate, and the plates were incubated at 28°C for 7 days. Negative control plates were prepared under the same conditions, with equal volumes of ethanol and water (1 mL) instead of the extracts. After 7 days, the diameter of the inhibition zones (halo of nongrowth) surrounding the wells was measured in millimeters. Additionally, a separate plate containing the standard antifungal agent (Nystatin) for *A. flavus* at a specific concentration was incubated at 37°C for 7 days as a positive control.

All the test steps were repeated three times for each sample, and the diameter of each of the inhibition zones was measured with a digital caliper.

#### 2.1.5. Determination of Minimum Inhibitory Concentration (MIC)

Following the preliminary screening, the MIC of the active antifungal agents was determined using the broth microdilution method, as outlined by the Clinical and Laboratory Standards Institute (CLSI) guidelines (document M38-A). The microplate dilution method was used to determine the MIC of the investigated compounds. A 7-day fungal culture was prepared in Sabouraud dextrose broth (SDB) medium, incubated at 28°C with shaking at 150 rpm. For the MIC determination, 100 *μ*L of SDB medium containing a fungal suspension equivalent to half-McFarland standard was added to each well of the microplate. Then, 100 *μ*L of pure propolis extract was added to the first well. Half of the solution was removed from this well and transferred to the next, continuing serial dilutions until well number 10. Each well's volume was adjusted to 100 *μ*L with the culture medium. Well Number 11 served as a positive control, containing 100 *μ*L of the culture medium with fungal spores, while well number 12 (negative control) contained 100 *μ*L of pure extract to account for the optical absorption of the extract itself. The microplate was incubated at 37°C for 48 h. These steps were also performed for the other tested compounds. Finally, the optical density of all wells was measured using a spectrophotometer.

#### 2.1.6. Determination of Minimum Fungicidal Concentration (MFC)

To determine the MFC of the studied compounds, all wells showing positive results for MIC were subcultured onto Sabouraud dextrose agar plates. The plates were sealed with parafilm and incubated for 72 h at 37°C. The lowest concentration at which no visible fungal growth occurred was recorded as the MFC of the extracts.

#### 2.1.7. Statistical Analysis

To measure the diameter of the inhibition zone, the data was statistically analyzed with the SAS software Version 9.1 using the ANOVA method. Differences between the means were considered significant at a *p* value of less than 0.05 using the Tukey method.

## 3. Results and Discussion

### 3.1. Characterization of the Nanocomposites

#### 3.1.1. X-Ray Diffraction (XRD) Analysis

**Figure 1:**
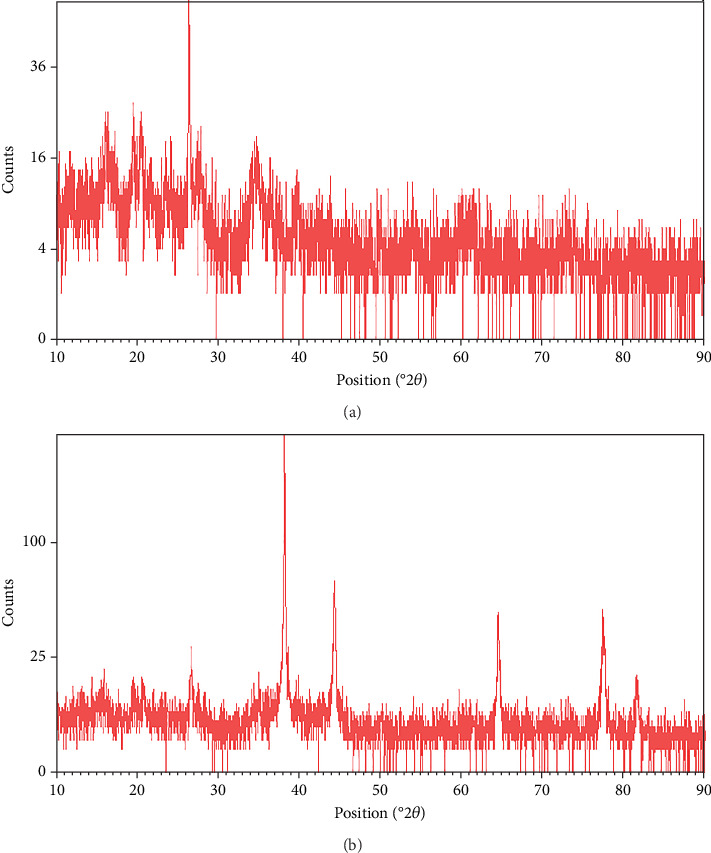
XRD pattern of (a) sepiolite/propolis and (b) sepiolite–Ag–propolis sample.

The XRD pattern of sepiolite/propolis is shown in Figure 1a. The diffraction pattern related to Pages 130, 060, 131, 260, 241, 080, 331, 341, 441, 371, 202, 541, and 791 which are, respectively, at angles 2*θ* = 12.03, 19.96, 20.83, 24.03, 25.43, 26.82, 28.19, 29.56, 33.51, 23. 35.85, 36.85, 40.08, and 60.10 are observed; based on comparison with the standard pattern of sepiolite (JCPDS Card No. 13-0595), it corresponds to the structure of sepiolite with orthorhombic unit cells [24]. In addition, the patterns in the angles of 44.01° (202), 58.22° (122), 62.28° (300), and 72.95° (128) related to calcite grid plates JCPDS Card No. 0586-05. Due to its amorphous structure, propolis does not show a peak in the XRD pattern and only leads to a decrease in the intensity of sepiolite peaks. Also, in Figure 1b, the peaks related to silver NPs at the angles of (111) 38.18°, (200) 44.25°, (220) 64.72°, (311) 77.40°, and (331) 45.83° are visible (JCPDS Card No. 01-087-0719), which indicates the successful synthesis of sepiolite–Ag–propolis nanocomposite.

#### 3.1.2. Fourier Transform Infrared Spectroscopy (FT-IR) Analysis

**Figure 2:**
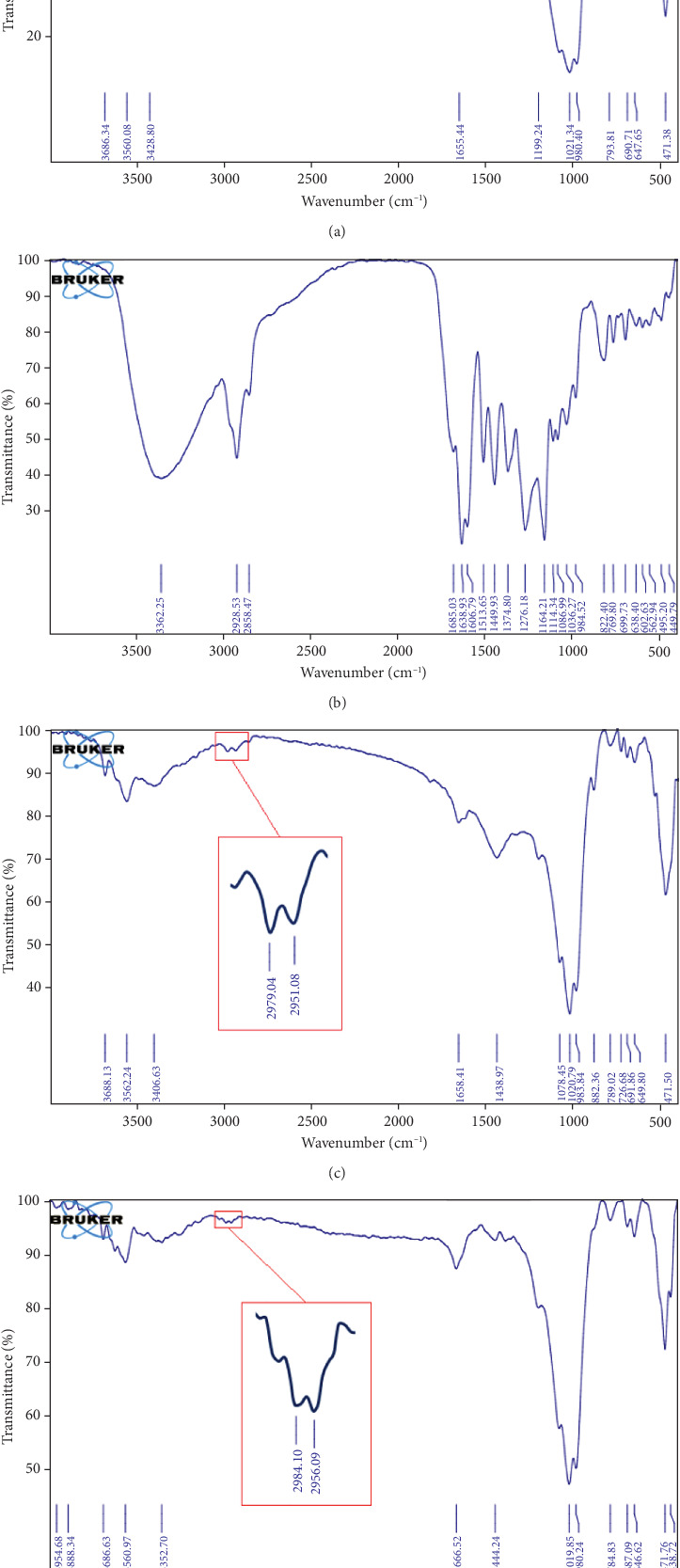
FTIR spectrum of (a) sepiolite, (b) propolis, (c) sepiolite/propolis, and (d) sepiolite–Ag–propolis nanocomposite.

The FT-IR spectra of sepiolite, propolis, sepiolite/propolis, and sepiolite–Ag–propolis nanocomposite are shown in Figures 2a, 2b, 2c, and 2d. In the sepiolite sample (Spectrum A), the absorption bands observed at, 3560, 3428, and 1655 cm^−1^ in the FT-IR spectrum are related to the hydroxy groups of water [25]. The characteristic peaks of Si–O–Si can be seen at 1021, 1199, and 980 cm^−1^ [26]. The absorption peak at 647 cm^−1^ indicates the bending vibrations of the Mg-OH bond [27]. The absorption band observed at 3362 cm^−1^ in the FT-IR spectrum of propolis (Spectrum B) is attributed to the hydroxy groups of its phenolic compounds [28]. The absorption bands in the regions of 1638, 1513, and 1449 cm^−1^ are related to the stretching vibrations of C=O and C-O bonds in flavonoid and polyol compounds [29]. The absorption band at 1164 cm^−1^ is related to C=C bond vibrations of alkenes, and the absorption peak at 1036 cm^−1^ region is related to C-O-C ether bond stretching vibrations [30]. Also, the absorption peaks ranging from 2558 to 2928 cm^−1^ are attributed to the stretching vibrations of C-H bonds in propolis [30]. In the sepiolite–propolis sample (Spectrum C), the presence of absorption peaks in the areas of 1438, 2951, and 2979 cm^−1^ indicates the successful coating of propolis on the sepiolite clay. For a better comparison, the FT-IR spectrum of sepiolite–Ag–propolis nanocomposite is also shown in Figure 2d.

#### 3.1.3. Transmission Electron Microscopy (TEM) Analysis

**Figure 3:**
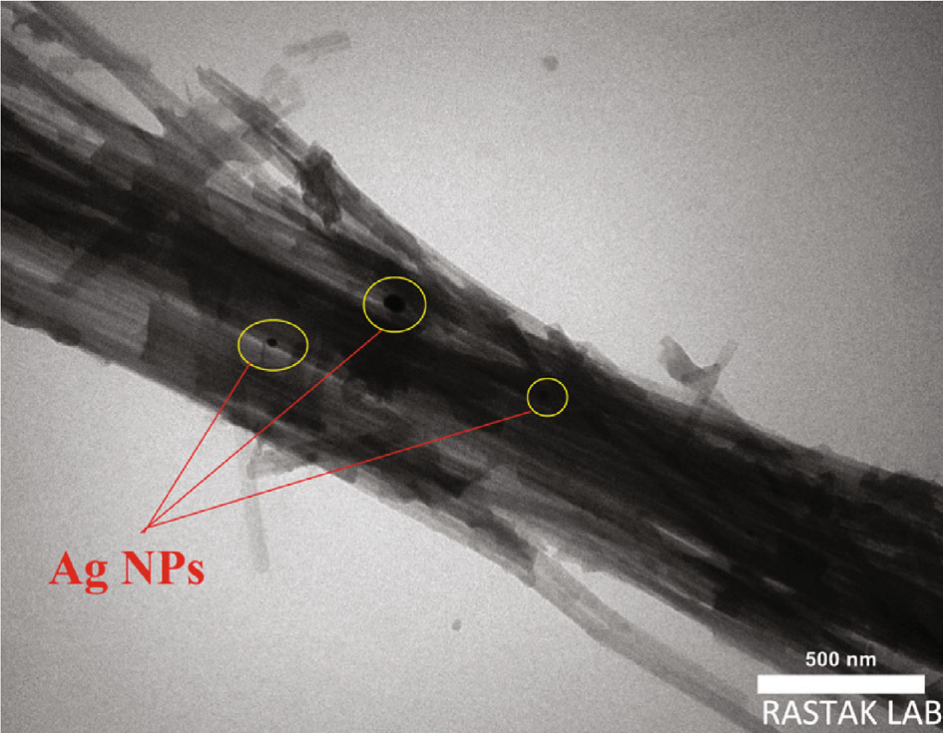
TEM image of sepiolite–Ag–propolis nanocomposite.

**Figure 4:**
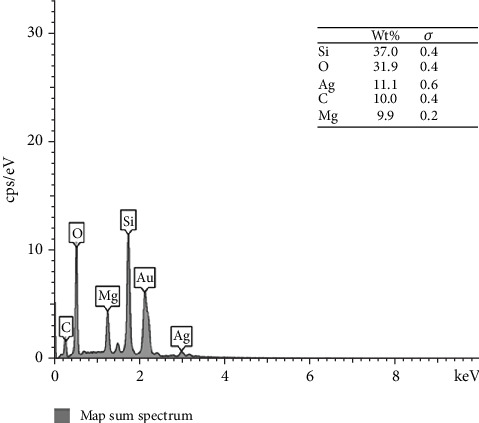
EDAX spectrum of sepiolite–Ag–propolis sample.

The morphology of the sepiolite–Ag–propolis composite was studied using TEM analysis, and the result is shown in Figure 3. According to the TEM image, the spherical shapes of Ag NPs are deposited on the needle-like structure of sepiolite support.  Figure 4 shows well-dispersed Ag NPs on the surface of sepiolite with a size of approximately 10–20 nm. Because this type of NP is homogeneous, it can contribute to increase the antimicrobial properties of this composite.

#### 3.1.4. Energy Dispersive X-Ray Analysis (EDAX) Analysis

The elemental composition of the synthesized sepiolite–Ag–propolis nanocomposite was investigated using EDAX. The EDAX spectrum (Figure 4) reveals the presence of characteristic peaks corresponding to the primary constituent elements of the nanocomposite. The dominant signals are attributed to Si (37.0 wt%) and O (31.9 wt%), which are consistent with the fundamental tetrahedral silicate structure of sepiolite [31]. The presence of Mg (9.9 wt%) further confirms the successful incorporation of sepiolite as the matrix material. Notably, a distinct Ag peak was observed, accounting for 11.1 wt% of the total composition, indicating the effective integration of Ag NPs within the sepiolite framework. The detection of C signals (10.0 wt%) provides supporting evidence for the presence of organic constituents from propolis in the nanocomposite structure. The observed weight percentages corroborate the successful synthesis of a multicomponent sepiolite–Ag–propolis nanocomposite. The relatively high silver content suggests a substantial loading of Ag NPs, which is particularly relevant for potential antimicrobial applications.

#### 3.1.5. Effect of Formulations on *A. flavus*

Ethanol extract of propolis and sepiolite/propolis nanocomposite and sepiolite–Ag–propolis nanocomposite had a significant amount of antifungal effects against *A. flavus*.  Table 1 shows these differences. As indicated in Table 1, the diameter of the obstruction halo of fungal growth has a direct relationship with the concentration of inhibitory compounds, and with their increase, the diameter of the growth halo has increased. The sepiolite–Ag–propolis nanocomposite displayed the highest antifungal activity, after that, the propolis–sepiolite composite. The propolis was found to have the smallest inhibitory halo of 10.27 mm at 750 mg/mL, and this value was rather inferior to nystatin (36.54 mm at 750 mg/mL). The incorporation of Ag NPs into the propolis–sepiolite matrix improved the antifungal properties (*p* < 0.05). This improvement is likely due to the compatibility of Ag NPs, which have a broad spectrum of antimicrobial activity.

The values of MIC and MFC of propolis, propolis–sepiolite, and sepiolite–Ag–propolis were established (Table 2). The MIC of propolis was 430 mg/mL which is more than the 265 mg/mL propolis–sepiolite and 220 mg/mL for sepiolite–Ag–propolis, indicating the effectiveness of sepiolite and Ag NPs towards enhancement of antifungal activity. The MFC of propolis, on the other hand, is 490 mg/mL, while the MFC for the composites propolis–sepiolite and sepiolite–Ag–propolis was 300.57 and 300 mg/mL, respectively. The standard antibiotic, nystatin, exhibited the lowest values of MIC at 50 mg/mL and MFC at 67.45 mg/mL.

In fungi and other organisms, primary metabolites are necessary for growth and reproduction, and secondary metabolites are formed at the end of growth and do not have specific importance in growth and metabolism. Typically, these compounds are formed when large amounts of primary metabolic precursors such as amino acid and pyruvate are produced and accumulated [32]. The pathogenicity of *A. flavus* is caused by the effects of aflatoxins. Many fungi produce toxic compounds called mycotoxins. Mycotoxins are secondary metabolites. Among mycotoxins, 14 types are carcinogenic. Among them, aflatoxins are the strongest compounds in terms of carcinogenesis. The growth of *Aspergillus flavus* is observed in foods such as animal and poultry feed, milk, wheat flour, soybeans, cheese, yogurt, and processed meats [33].

So far, many studies have been conducted on chemicals such as sulfites and bisulfites to target aflatoxins, which are toxins secreted by *Aspergillus* species in food [33]. Common plant antioxidants include tocopherols, flavonoids, and related compounds such as coumarins, cinnamic acid derivatives, phenolic diterpenes, and phenolic acid. Propolis consists of a wide variety of chemical compounds such as resins, aromatic balms, waxes, essential oils, pollen grains, and flavonoids [34]. In general, the activity against microorganisms is more related to the synergistic effect of flavonoids than to the action of each of them separately, such as galanga and pinocembrin. The process of antifungal effect of these compounds due to antioxidant compounds can be through damage to the mitochondrial DNA, damage to the cell wall, and finally the death of the microorganism [32, 35]. The presence of flavonoids and other phenolic compounds in propolis extract has been reported, and the antibacterial and antifungal properties of this substance have been investigated and confirmed in many studies [32, 36, 37].

The antifungal activity of propolis was investigated in sensitivity tests on 80 *Candida* yeast strains, 20 *Candida albicans* strains, 20 *Candida tropicalis* strains, 20 *Candida krusei* strains, and 15 *Candida Guillermondi* strains. The results demonstrated significant antifungal activity against all tested strains. For example, patients treated with hydroalcoholic propolis extract showed a noticeable reduction in the number of *Candida guilliermondii* strains [38]. Also, various investigations have shown that propolis extract is much more effective on Gram-positive bacteria than Gram-negative bacteria. This difference in effect is considered to be due to the difference in the cell wall structure of Gram-positive and negative bacteria [39]. On the other hand, the plant source, the season of propolis production, and the type of solvent used also have a significant effect on the antibacterial effects of propolis because organic solvents release more compounds from propolis, and with these solvents, many antibacterial effects are obtained [40].

Findings from this study showed that propolis-based nanocomposites have great potential for antifungal applications, especially against *Aspergillus flavus*. In addition, the incorporation of sepiolite and Ag NPs into propolis gave a greater antifungal action, as can be shown by the larger halos of inhibition and lower MIC and MFC values than those obtained using only propolis. This, therefore, enhances the antifungal efficacy through some sort of synergy between propolis, which is known to have natural antimicrobial properties, and Ag NPs themselves, which are renowned for their antimicrobial potency.

EDAX and FT-IR analyses confirmed the successful incorporation of propolis into the sepiolite matrix. The presence of the C element in the EDAX spectrum along with the slight shifts in the FT-IR peaks confirms the successful loading of propolis onto sepiolite clay, and Ag NPs were well dispersed. XRD and TEM analyses further confirmed the presence of Ag NPs on the surface of propolis–sepiolite nanocomposite.

The better performance of the sepiolite–Ag–propolis nanocomposite than the single components indicates that the nanocomposite formulation offers more effective and sustained antifungal action. Sepiolite may act as a carrier, allowing a high surface area for the dispersion of Ag NPs and stabilizing the molecules of propolis. This combination enhances the bioavailability of the active components, allowing for more efficient fungal inhibition.

On the other hand, propolis exhibited somewhat lower antifungal activity, which might be due to its limited capabilities in infiltrating fungal cell membranes. Ag NPs added in this case are known for disruption of microbial membranes, with the generation of reactive oxygen species, which may enhance the overall antifungal effect of the nanocomposite.

In previous reports, the synergistic activity of the combination of chitosan and propolis extract has been investigated. Torlak and Sert [41] reported that the antimicrobial activity of chitosan-based formulations was improved by the addition of propolis extract. When using them, bacterial growth was 3 log CFU, while when combined with propolis at 10% concentration, it was 1 log CFU. Also, Cortes-Higareda et al. [32] identified that the NPs of chitosan and propolis and the extract of propolis at the highest concentration of 40% exerted a notable inhibition on the spore germination and, principally, on the aflatoxin production of *A. flavus*. Likewise, Mattiuz et al. [42] reported that propolis (3:7 v/v) reduced the growth of the plant pathogenic fungus *Diplodia seriata* by 75%, but the inhibition was 100% when using a combination of chitosan and propolis (1.1 w/v). Also, Tharani et al. [43] discussed Ag NPs synthesized using *Terminalia chebula* and showed their antimicrobial properties and low toxicity. This aligns with our findings on the enhanced antifungal effect of the sepiolite–Ag–propolis nanocomposite. Ag NPs can increase the effectiveness of natural compounds such as propolis in microbial inhibition by disrupting microbial cell walls [43].

Because sepiolite has special pore structures, rich surface groups, high specific surface area, and better ion exchange capacity, it is possible to make antimicrobial nanocomposites and functional hybrid materials. The silanol groups on the external surface of this 1D clay mineral and its surface electric charge play an important role in the interaction with various antimicrobial agents, and the presence of structural cavities (tunnels and channels) allows small molecules to immobilize inside them. In addition, the characteristics of nontoxicity, biocompatibility, and compatibility with the environment have made 1D clay mineral and its derived composites widely used in human health-related fields.

Moreover, based on the findings of Shanmugam et al. [44], who investigated the potential of natural extract nanocomposites as antimicrobial agents, especially, in food safety, combining sepiolite with propolis and Ag NPs can be an innovative and safe approach for agricultural applications. Therefore, antimicrobial nanocomposites can be used in various fields, such as food packaging and feed additives for animal health, and become innovative antimicrobial nanomaterials to inhibit pathogenic fungus.

## 4. Conclusion

In this study, it was found that the propolis extract and its components combined with nanocomposites of sepiolite and Ag NPs significantly prevent the spore germination of *A. flavus*. Nevertheless, since these results are under carefully controlled conditions, further research should be extended to in vivo proposals on different agricultural products affected by this fungus.

## Figures and Tables

**Table 1:** Inhibition zone diameters (millimeters) of different concentrations of propolis extracts and propolis nanocomposites against *A. flavus* in the agar well diffusion method.

**Dilution of the extract (mg/mL)**	**Propolis**	**Sepiolite–propolis**	**Sepiolite–Ag–propolis**	**Nystatin**
50	7.69 ± 0.03^cC^	9.86 ± 0.05^bcBC^	10.05 ± 0.02^cB^	25.21 ± 0.02^bA^
100	8.9 ± 0.07^bC^	10.35 ± 0.04^bB^	10.87 ± 0.05^bB^	27.32 ± 0.04^bA^
250	9.1 ± 0.02^bC^	10.68 ± 0.06^bBC^	11.1 ± 0.04^bB^	30.78 ± 0.03^abA^
500	10.24 ± 0.05^aC^	11.55 ± 0.02^aB^	11.55 ± 0.05^aB^	34.2 ± 0.04^aA^
750	10.27 ± 0.05^aC^	11.69 ± 0.04^aB^	12.03 ± 0.03^aB^	36.54 ± 0.05^aA^

*Note:* In each column, the mean with the same lowercase letter is not significantly different at the 5% level. In each row, the mean with the same uppercase letter is not significantly different at the 5% level.

**Table 2:** Minimum inhibitory concentration (MIC) and minimum fungicidal concentration (MFC) of propolis extract and propolis nanocomposites for *A. flavus*.

**Extract**	**MIC**	**MFC**
Propolis	430 ± 32.65	490 ± 25.00
Sepiolite–propolis	265 ± 10.5	300.57 ± 40.2
Sepiolite–Ag–propolis	220 ± 20.2	300 ± 65.00
Nystatin	50 ± 0.00	67.45 ± 0.5

## Data Availability

The data used to support the findings of this study are available from the corresponding author upon request.
